# The correlation between eBird community science and weather surveillance radar‐based estimates of migration phenology

**DOI:** 10.1111/geb.13567

**Published:** 2022-07-13

**Authors:** Elaina K. Haas, Frank A. La Sorte, Hanna M. McCaslin, Maria C. T. D. Belotti, Kyle G. Horton

**Affiliations:** ^1^ Department of Fish, Wildlife, and Conservation Biology Colorado State University Fort Collins Colorado USA; ^2^ Cornell Lab of Ornithology Cornell University Ithaca New York USA

**Keywords:** bird migration, citizen science, community science, eBird, phenology, radar remote sensing

## Abstract

**Aim:**

Measuring avian migration can prove challenging given the spatial scope and the diversity of species involved. No one monitoring technique provides all the pertinent measures needed to capture this macroscale phenomenon – emphasizing the need for data integration. Migration phenology is a key metric characterizing large‐scale migration dynamics and has been successfully quantified using weather surveillance radar (WSR) data and community science observations. Separately, both platforms have their limitations and measure different aspects of bird migration. We sought to make a formal comparison of the migration phenology estimates derived from WSR and eBird data – of which we predict a positive correlation.

**Location:**

Contiguous United States.

**Time period:**

2002–2018.

**Major taxa studied:**

Migratory birds.

**Methods:**

We estimated spring and autumn migration phenology at 143 WSR stations aggregated over a 17‐year period (2002–2018), which we contrast with eBird‐based estimates of spring and autumn migration phenology for 293 nocturnally migrating bird species at the 143 WSR stations. We compared phenology metrics derived from all species and WSR stations combined, for species in three taxonomic orders (Anseriformes, Charadriiformes and Passeriformes), and for WSR stations in three North American migration flyways (western, central and eastern).

**Results:**

We found positive correlations between WSR and eBird‐based estimates of migration phenology and differences in the strength of correlations among taxonomic orders and migration flyways. The correlations were stronger during spring migration, for Passeriformes, and generally for WSR stations in the eastern flyway. Autumn migration showed weaker correlation, and in Anseriformes correlations were weakest overall. Lastly, eBird‐based estimates slightly preceded those derived from WSR in the spring, but trailed WSR in the autumn, suggesting that the two data sources measure different components of migration phenology.

**Main conclusions:**

We highlight the complementarity of these two approaches, but also reveal strong taxonomic and geographic differences in the relationships between the platforms.

## 1 INTRODUCTION

Understanding the broad‐scale timing of life history events such as breeding, or the progression of seasonal migration is critical in understanding how organisms respond to changing environments (Visser & Both, 2005). While achieving a macrosystem view of phenology can be challenging, particularly when working with diverse ecological systems (e.g., high species richness), tools and techniques are available for such monitoring approaches. However, understanding the biases and limitations of these monitoring approaches is increasingly important, particularly when exploring issues related to climate change (Youngflesh et al., 2021), applied conservation (Horton et al., 2021), and human–wildlife conflicts (Ruiz‐Gutierrez et al., 2021). Phenology is of broad interest across taxonomic groups (Cohen et al., 2018), and migration phenology of birds can serve as bellwethers for potential trophic mismatches and population declines in the face of recent changes in climate (Both et al., 2006; Charmantier & Gienapp, 2014; Hurlbert & Liang, 2012). However, quantifying and understanding these patterns at broad scales remains challenging.

Each spring and autumn, billions of migratory birds travel hundreds to thousands of kilometres between their breeding and non‐breeding grounds, traversing hemispheres to track seasonal productivity (Dokter et al., 2018; La Sorte, Fink, Hochachka, DeLong, & Kelling, 2014; La Sorte & Graham, 2021; Youngflesh et al., 2021). Given the spatial and temporal scale of movements made by migratory birds, comprehensive efforts to monitor annual avian migration represent a continual interest in macrosystem ecology. A variety of empirical resources are available, from bird‐banding data (Covino et al., 2020) to tracking technologies, (Taylor et al., 2017), but also larger‐scale measurements by radar (Dokter et al., 2018) and observations provided by community (citizen) science platforms (La Sorte et al., 2013; Sullivan et al., 2014).

While weather surveillance radar (WSR) provides large spatial coverage (e.g., continental scale) and collects measurements that directly capture nocturnal in‐flight migration, this tool often falls short in providing species‐specific observations (Kelly & Horton, 2016). Conversely, the strength of eBird community science data lies in the species‐specific nature of the diurnal observations, although translating those observations to absolute abundance is difficult (Johnston et al., 2015). Additionally, like nearly all diurnal, ground‐based observations of migratory birds, it is challenging to disentangle the migratory status of the individuals observed – especially for species where individuals of varying life history stages overlap geographically (Callaghan & Gawlik, 2015). Across these two platforms, limitations of one method may be compensated by the other, and likely the integration of these two pathways would yield a more comprehensive monitoring approach (Horton et al., 2018; Horton, Van Doren, et al., 2019; Nilsson et al., 2021; Weisshaupt et al., 2021). Community science (e.g., eBird) fills the species‐specific gaps in radar and radar addresses the inability of community science to delineate periods of active migration or estimate absolute abundance. However, it remains unclear how phenology metrics compare across the two platforms.

The 143 WSR stations located across the contiguous United States provide nearly complete aerial coverage of the country, spanning upwards of 20° of latitude. WSR stations measure the intensity of nocturnal migration via radar reflectivity, which can be used to quantify nightly, and by extension, seasonal passage of migrants. Because the vast majority of migrants fly at night (~80% of migratory species), nocturnal WSR scans capture the bulk of migratory passage (Horton, Nilsson, et al., 2019). Parallel efforts to monitor avian migration phenology have been accomplished by leveraging WSR to investigate phenological shifts on a continental scale (Horton et al., 2020) and through occurrence and abundance observations from the eBird community science database (Hurlbert & Liang, 2012; La Sorte & Graham, 2021; Mayor et al., 2017; Youngflesh et al., 2021). Although studies have examined broad‐scale migration phenology using these two data resources, these approaches have not been paired to examine similarities and differences in their estimates.

To this end, we sought to make a formal comparison of the migration phenology estimates derived from WSR and eBird data – of which we predict a positive correlation. In addition to measuring correlation, we also explore the difference in the magnitude of phenology estimates between platforms. For instance, does one platform consistently lead to earlier or later phenology estimates? We examine how the correspondence between the two platforms varies between spring and autumn migration, among dominant species in three taxonomic orders (Anseriformes, Charadriiformes and Passeriformes), and among WSR stations in three North American migration flyways (western, central and eastern; La Sorte, Fink, Hochachka, Farnsworth, et al., 2014). Because birds detected and measured at WSR stations in North America are largely composed of Passeriformes (Dokter et al., 2018; Horton, Nilsson, et al., 2019; Nilsson et al., 2018), we expect eBird migration phenology estimates derived for Passeriformes to be more strongly correlated with WSR estimates. Finally, we predict the correlation between the two approaches will be strongest in the eastern flyway, which contains among the three flyways the highest proportion of migrating Passeriformes (La Sorte, Fink, Hochachka, Farnsworth, et al., 2014).

## 2 METHODS

In what follows, we describe in detail the data extraction and processing steps required to obtain our target phenology metric for both weather surveillance radar (WSR) and eBird data. For the purposes of this study, we opted to use the first date when mean abundance reaches half of its peak value as an estimate of seasonal migration phenology (Youngflesh et al., 2021). This choice reflects the need to select a robust metric capable of capturing the full range of species‐specific patterns of migration across a large spatial extent. Particularly in the case of eBird data, diurnal ground observations make it challenging to distinguish whether each observed individual is actively migrating or whether it is residing within its breeding or non‐breeding grounds. On Supporting Information Figure S1, we demonstrate this variation both conceptually and for the specific case of the gray catbird (*Dumetella carolinensis*) in three separate locations of its annual geographic distribution.

### 2.1 Radar

We used level II data from 143 WSR stations to quantify avian migration passage in both spring (1 March–15 June) and autumn (1 August–15 November) between the years 2002 and 2018. These observations were taken between dusk and dawn, capturing the movement of nocturnal migrants across North America at the five lowest elevation scans recorded by the radar (~0.5°, 1.5°, 2.5°, 3.5° and 4.5°) at 30‐min intervals. These data were acquired through the Amazon Web Service portal (https://s3.amazonaws.com/noaa‐nexrad‐level2/index.html). From the raw data, we isolated and preserved reflectivity classified as biological and removed any precipitation or other reflective clutter. We used MistNet, a convolutional neural network, to classify raw radar data before we aggregated or summarized any radar data (Lin et al., 2019).

From precipitation‐free scans, we built profiles of vertical activity from 0 to 3,000 m above ground level at 100‐m intervals, capturing migration intensity, via reflectivity, and migration speed and direction, via radial velocity. We extracted radar measures from a circular buffer centred at each WSR station with a radius of 5 to 37.5 km (i.e., a 37.5‐km buffer with a 5‐km buffer removed from the centre). As the radar beam propagates from the point of origin, the beam broadens, reducing spatial resolution, and additionally, distant volumes can show greater uncertainty in beam height estimates due to non‐standard refraction – for this reason, we use a narrower range of data, 37.5 km (Farnsworth et al., 2016).

**FIGURE 1:**
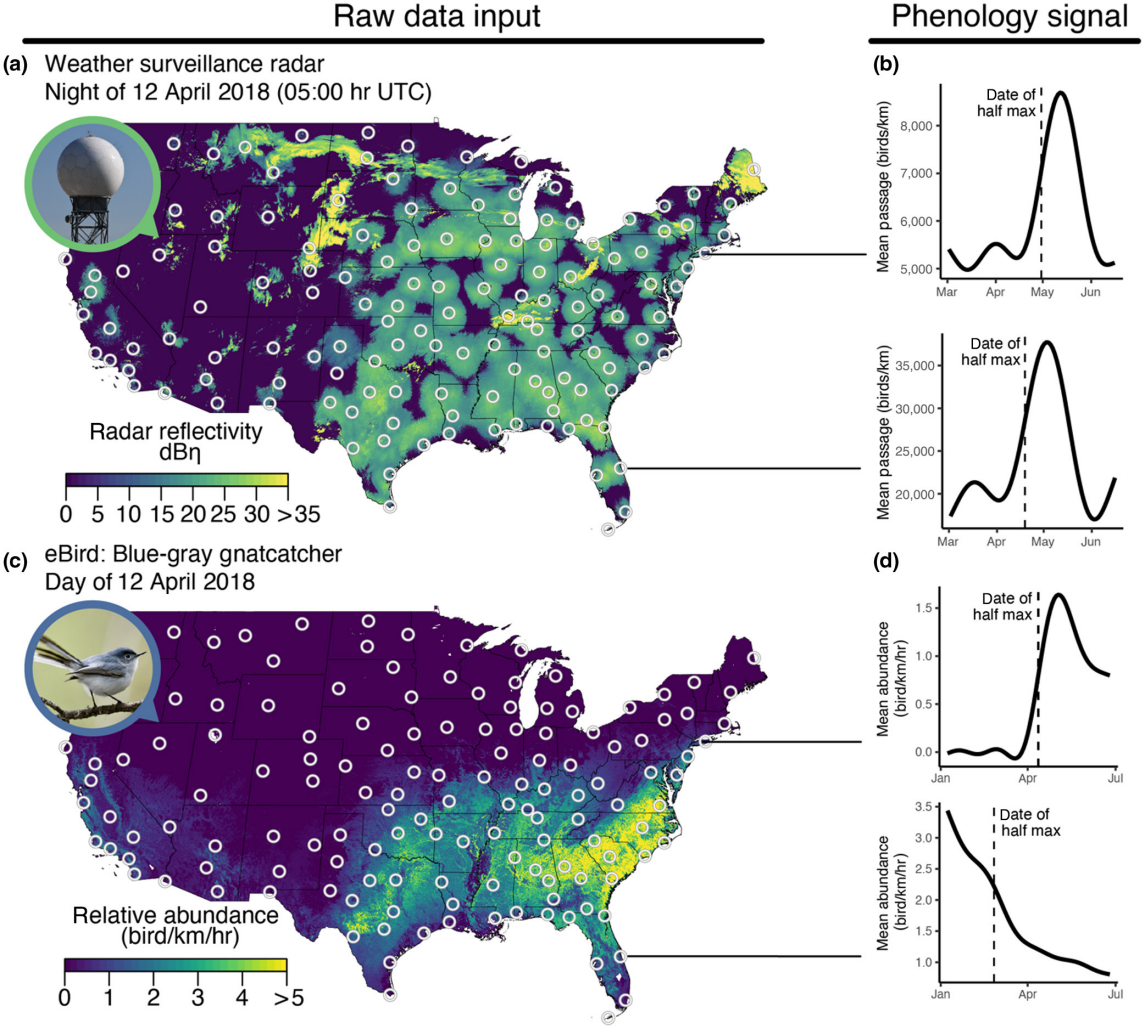
(a) Mosaic of radar reflectivity using data from 143 weather surveillance radar (WSR) stations (white circles) for the night of 12 April 2018, at approximately 05:00 hr UTC, with biological reflectivity shown in green, and weather contamination (i.e., precipitation) shown in yellow. (b) The date of half‐max passage estimated from the predicted curves of nightly migration passage rates. (c) Estimated relative abundance (no. birds/km/hr) for the blue‐gray gnatcatcher (*Polioptila caerulea*) during the week of 12 April 2018, overlaid with the locations of the 143 WSR stations (white circles). (d) Mean relative abundance of the blue‐gray gnatcatcher during spring migration at a southern WSR station (KMLB in Florida) and at a northern WSR station (KOKX in New York), demonstrating the northward movement in migration and their respective dates of half‐max relative abundance

For each radar sample taken every 30 min, we first converted radar reflectivity factor (dBZ) to radar reflectivity (η) following Chilson et al. (2012). We used reflectivity as a measure of migration intensity and multiplied it by groundspeed to obtain the rate of migratory passage at each of the 100‐m height intervals, and summed this rate through the night to yield a nightly measure of migrant passage; see Horton, Van Doren, et al. (2019) for additional details. For each WSR station, we then fit season‐specific generalized additive models (GAMs) to the time series of nightly passage rates (response) and ordinal date as a smooth predictor term across all years. Using the model to predict daily passage rates, we first determined the level of maximum seasonal passage. We then estimated the date of half‐max during spring and autumn, defined as the first date prior to the date of maximum passage that corresponded to half of the maximum passage (Figure 1b). In each model, we included year as a random effect.

### 2.2 eBird

eBird is a community science platform operated by the Cornell Lab of Ornithology designed to aid bird watchers in tracking and cataloguing bird sightings, while contributing to a global network of data that informs biodiversity and conservation science (Sullivan et al., 2014). Entries are compiled into a single online database, screened by predetermined filters, and when necessary, vetted by expert regional moderators. To date, eBird is the largest ecological community science database in the world, documenting the occurrence and abundance of thousands of bird species across the globe.

Unlike traditional large‐scale bird occurrence databases, eBird is a semi‐structured ‘big data’ resource. As such, statistical models are needed to standardize estimates of occurrence and abundance across space and time based on variation in observer skill, observer effort, habitat, and geography (Johnston et al., 2021). In our eBird analysis, we used weekly relative abundance estimates generated by adaptive spatio‐temporal exploratory models (AdaSTEMs), which are processed and hosted by the Cornell Lab of Ornithology (Fink et al., 2013; Fink, Auer, Johnston, Strimas‐Mackey, et al., 2020). AdaSTEM uses training observations from 1 January 2014 to 31 December 2018 and a total of 88 predictor variables from three classes: observation‐effort (*n* = 6), dimensions of time (*n* = 3), and environmental variables (*n* = 79). We acquired weekly AdaSTEM estimates of relative abundance (*abundance_median*) for a total of 610 species at a 2.96 × 2.96 km spatial resolution for the year 2018 within the Western Hemisphere using the R package *ebirdst* (Auer et al., 2020). We retained relative abundance estimates for 293 nocturnally migrating bird species (Horton, Nilsson, et al., 2019). We selected nocturnally migrating species because the bulk of the migratory species in North America migrate at night. Abundance estimates were denoted as *relative* because they do not capture the full complexity of species‐specific absolute detection probabilities, and thus serve as an index of total count of individuals detected by an expert observer at the optimal time of day and distance to maximize detection of the species (Fink, Auer, Johnston, Ruiz‐Gutierrez, et al., 2020). We extracted weekly estimates of relative abundance for each of the 293 species within a radius of 37.5 km centred at the 143 WSR stations. We then averaged these values for each species, week, and WSR station.

We estimated spring and autumn migration phenology for each species and WSR station using a generalized additive model (Wood, 2011) with weekly estimates of relative abundance as the response and ordinal date as a smooth predictor term. GAMs were fit separately for each species and season, with spring migration GAMs fit from 4 January to 28 June and autumn migration GAMs fit from 6 July to 28 December. AdaSTEM relative abundance estimates are made for the midpoints of each week. The beginning and end of our two seasonal migration periods reflect this distinction. We extracted GAM predicted daily relative abundance estimates for each species, season, and WSR station. We then estimated the date of half‐max during spring and autumn migration, defined as the first date prior to the date of maximum relative abundance that corresponded to half of the maximum predicted relative abundance (Figure 1). If relative abundance estimates peaked early in the season (e.g., before ordinal day 10 in spring and before ordinal day 185 in autumn, Figure 1d), we selected the first date after the date of maximum relative abundance that corresponded to half of the maximum predicted relative abundance. We only included half‐max estimates if the adjusted *R* ^2^ from the generalized additive model was greater than 25%. Lastly, to ensure that the WSR station had the potential to detect each species while in active migration, we restricted half‐max estimates to the periods between 1 March and 15 June during spring migration and between 1 August and 15 November during autumn migration.

In total, all 293 nocturnally migrating species met our model fit criteria (e.g., *R* ^2^ from GAM > 25%) in spring and 279 in the autumn. In the spring, the 293 species were distributed among 12 taxonomic orders. Passeriformes made up most (57%) of the 293 species, followed by Charadriiformes (16%) and Anseriformes (13%). The remaining nine orders individually accounted for up to 4% of the species (total of 14%). For the autumn migration analysis, we retained 279 nocturnally migrating species distributed between 11 taxonomic orders. Passeriformes totalled 57% of the 279 species, again followed by Charadriiformes (16%) and Anseriformes (13%). The remaining eight orders together made up the remaining 14%. The next highest order not included was Pelecaniformes in both spring and autumn, which made up only 3 and 4% of diversity, respectively. For this reason, we saw it as a breakpoint in the described results.

### 2.3 Data analysis

We calculated Pearson's correlation coefficient to assess the correspondence between the half‐max passage rate (WSR) phenology estimates and the average half‐max (eBird) migration phenology estimates for each species. Specifically, the seasonal analyses were comprised of a single phenology metric (ordinal date) per data source per radar station (*n* = 143). We summarized the difference between the WSR and eBird migration phenology estimates by subtracting the average half‐max (eBird) day of the year for each species from the half‐max passage rate (WSR) day of the year. Negative values indicated WSR estimated earlier migration phenology than eBird, and positive values indicated eBird estimated earlier migration phenology than WSR. From the eBird standpoint, in addition to examining these relationships using the average of all nocturnally migrating bird species at the 143 WSR radar station locations, we categorized the half‐max phenology estimates into the three most species rich orders (Anseriformes, *n* = 38; Charadriiformes, *n* = 47; and Passeriformes, *n* = 167) and the WSR stations into one of the three North American migration flyways (western, *n* = 40; central, *n* = 42; and eastern, *n* = 61). We defined the western flyway as the contiguous United States west of 103° west longitude, the central flyway as the contiguous United States between 103° and 90° west longitude, and the eastern flyway as the contiguous United States east of 90° west longitude (La Sorte, Fink, Hochachka, Farnsworth, et al., 2014). We used one‐way ANOVAs to examine statistical differences in means of species richness and magnitude offset across flyways, and when appropriate, Tukey honestly significant difference (HSD) post‐hoc tests to identify specific pairwise differences. All analyses and figures were generated using R version 4.0.2 (R Core Team, 2020). We used the *mgcv* package to implement the generalized additive models (Wood, 2011).

## 3 RESULTS

We compiled WSR spring and autumn migration phenology estimates from 2,225 year–site spring replicates and 2,102 year–site autumn replicates from 2002 to 2018. Spring WSR estimates of half‐max dates occurred earlier at lower latitudes and later at higher latitudes, paralleling the northward movement of migrants, with the opposite observed in the autumn as migrants travelled south. Similar latitudinal trends were evident in the spring with eBird estimates of half‐max.

### 3.1 Species richness

**FIGURE 2:**
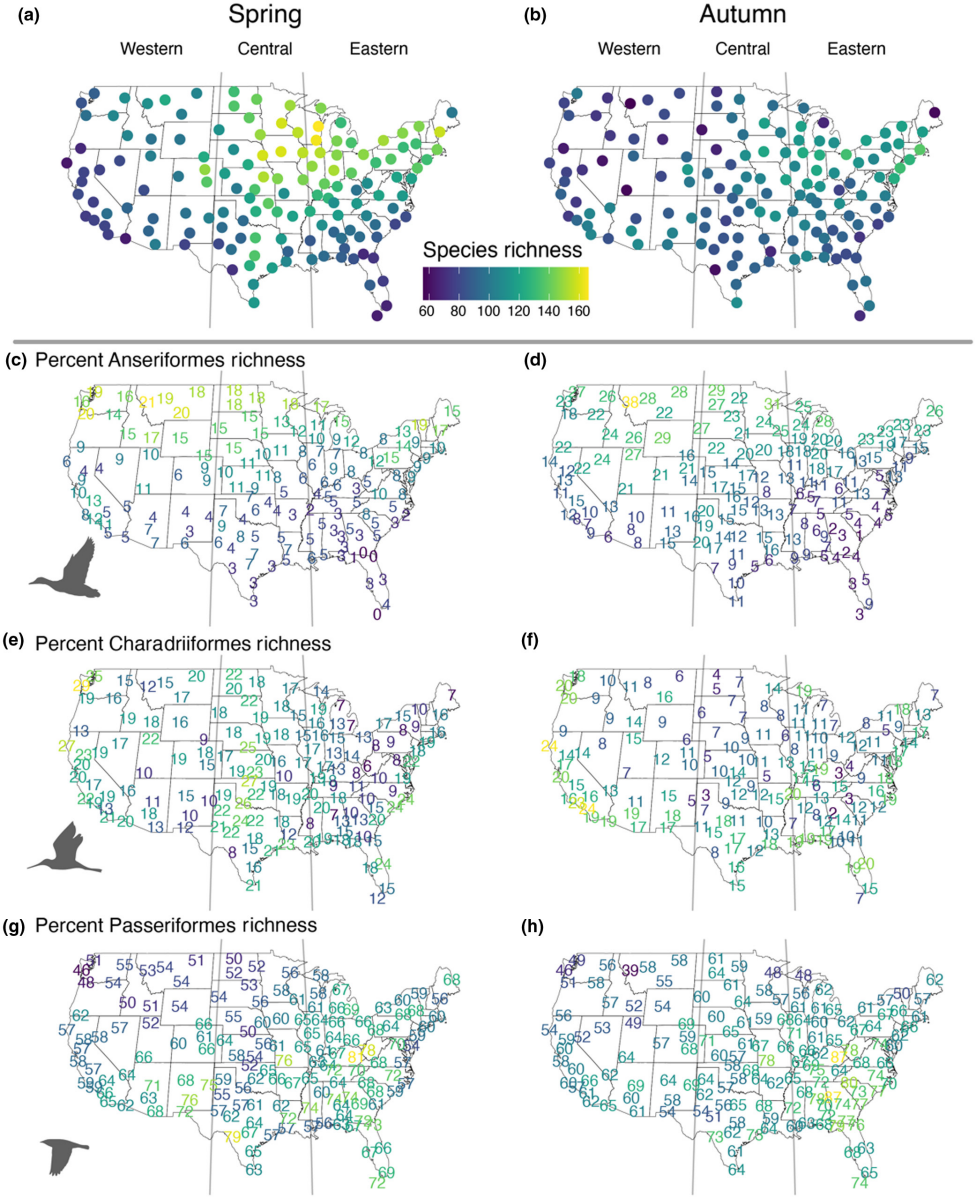
Species richness of nocturnally migrating birds estimated at 143 weather surveillance radar (WSR) stations using eBird data during (a) spring and (b) autumn migration. A total of 293 nocturnally migrating bird species were considered in our analyses. The percentages of Anseriformes (c, d; *n* = 38), Charadriiformes (e, f; *n* = 47) and Passeriformes (g, h; *n* = 167) species richness estimated at 143 WSR stations using eBird data are shown. These values may not sum to 100 given species in other orders, that were not included in analysis, may be present. The vertical grey lines delineate the boundaries between the western, central and eastern North American migration flyways

**FIGURE 3:**
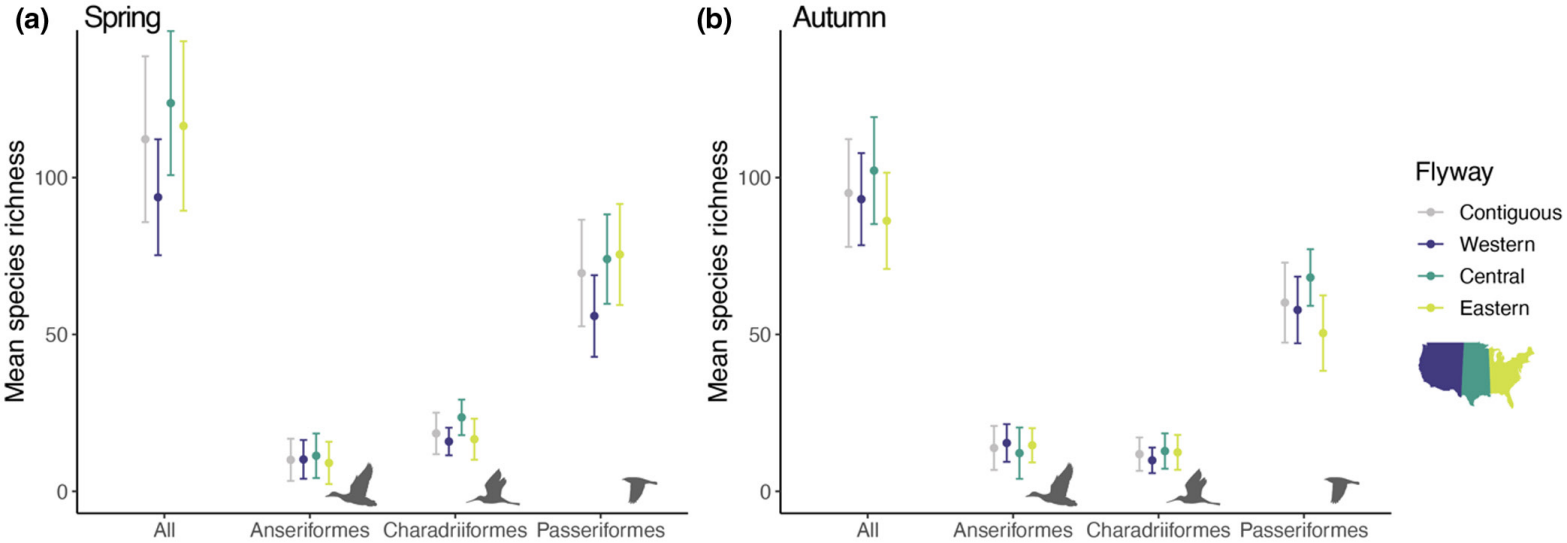
Mean (± *SD*) species richness of nocturnally migrating birds estimated at 143 weather surveillance radar (WSR) stations using eBird data during (a) spring and (b) autumn migration. A total of 293 nocturnally migrating bird species were considered in the analysis

For all species combined, the 143 WSR stations on average contained 112.2 ± 26.4 (± *SD*) species during spring migration (Figures 2a and 3a) and 95.0 ± 17.2 (± *SD*) species during autumn migration (Figures 2b and 3b). The 143 stations contained significantly more species during spring migration (Figures 2a and 3a) compared to autumn migration (17.1 ± 21.0; paired *t* test, *t*_142_ = 9.76, *p* < .001; Figures 2b and 3b). Species richness at the WSR stations differed significantly on average among the three flyways during spring (*F*_2,140_ = 18.09, *p* < .001; Figures 2a and 3a) and autumn migration (*F*_2,140_ = 18.09, *p* < .001; Figures 2b and 3b). During spring migration, Tukey HSD post‐hoc tests revealed significant differences in species richness between the western and central flyways during spring migration (mean difference = 30.0 species, *p* < .001; Figures 2a and 3a) and western and eastern flyways (mean difference = 22.7 species, *p* < .001; Figures 2a and 3a). During autumn migration, Tukey HSD post‐hoc tests did not reveal any significant pairwise differences.

The 40 WSR stations in the western flyway contained 93.7 ± 18.5 (± *SD*) species on average during spring migration (Figures 2a and 3a) and 86.2 ± 15.3 (± *SD*) species on average during autumn migration (Figures 2b and 3b). The 42 WSR stations in the central flyway contained 123.7 ± 23.0 (± *SD*) species on average during spring migration (Figures 2a and 3a) and 93.1 ± 14.7 (± *SD*) species on average during autumn migration (Figures 2b and 3b). The 61 WSR stations in the eastern flyway contained 116.4 ± 27.0 (± *SD*) species on average during spring migration (Figures 2a and 3a) and 102.2 ± 17.0 (± *SD*) species on average during autumn migration (Figures 2b and 3b). Of the three most species rich orders, Passeriformes contained the highest number of species across WSR stations on average (spring = 69.6 ± 17.0, autumn = 60.2 ± 12.7, mean ± *SD*; Figures 2g,h and 3) and Charadriiformes (spring = 18.5 ± 6.6, autumn = 11.9 ± 5.3, mean ± *SD*; Figures 2e,f and 3) showed lower species richness on average.

### 3.2 Correlation in migration phenology indices

**FIGURE 4:**
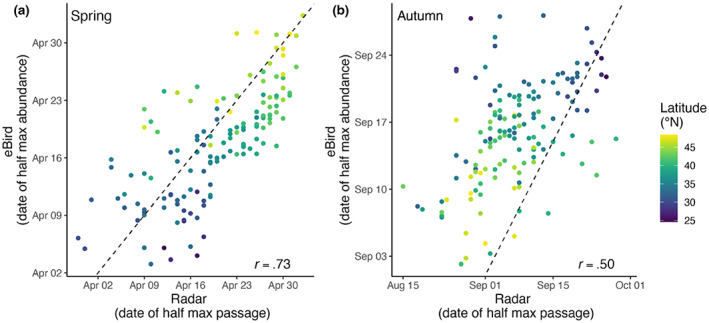
The relationship between ordinal date of half‐max passage estimated at 143 weather surveillance radar (WSR) stations and ordinal date of half‐max relative abundance estimated using eBird data during (a) spring and (b) autumn migration. Each of the 143 WSR sites is represented by a point on the plot, with their respective latitudes distinguished by a colour gradient. Pearson's correlation coefficient is shown in the bottom right of each seasonal plot and the dashed line shows the 1:1 relationship (identity line)

**FIGURE 5:**
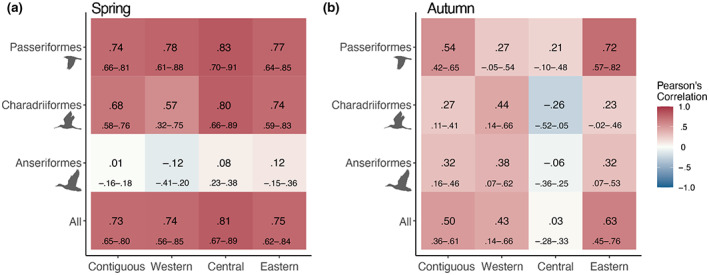
Mean Pearson's correlation coefficients with 95% confidence intervals between weather surveillance radar (WSR) and eBird‐based estimates of migration phenology at 143 WSR stations during (a) spring and (b) autumn migration. Correlations are shown for WSR stations within the contiguous United States and three North American migration flyways (western, *n* = 40; central, *n* = 42; and eastern; *n* = 61) and across all nocturnal migrant species and species in three taxonomic orders (Anseriformes, *n* = 38; Charadriiformes, *n* = 47; and Passeriformes, *n* = 167)

For all species combined across the 143 WSR stations, we found positive correlations between the WSR and eBird based estimates of migration phenology during spring (*r* = .73, *df* = 141, *p*‐value < .001, Confidence Interval (CI): .65–.80; Figure 4a) and autumn migration (*r* = .50, *df* = 141, *p*‐value < .001, CI: .36–.61; Figure 4b). Among species in the three taxonomic orders, Passeriformes had the strongest correlations between phenology indices during both spring (*r* = .74, *p*‐value < .001, CI: .66–.81; Figure 5a) and autumn migration (*r* = .54, *p*‐value < .001, CI: .42–.65, Figure 5b). During spring migration, correlation strength was intermediate for Charadriiformes (*r* = .68, *p*‐value < .001, CI: .58–.76), and no correlation was found for Anseriformes (*r* = .01, *p*‐value = .897, CI: −.16–.18) (Figure 5a). During autumn migration, correlation strength was intermediate for Anseriformes (*r* = .32, *p*‐value < .001, CI: .16–.46) and weakest for Charadriiformes (*r* = .27, *p*‐value = .001, CI: .11–.41) (Figure 5b). Across flyways, correlation strength wasn't markedly different during spring migration, although the western and eastern flyways showed stronger relationships during autumn migration as compared with the central flyway (Figure 5).

### 3.3 Differences in the magnitude of the two phenology indices

**FIGURE 6:**
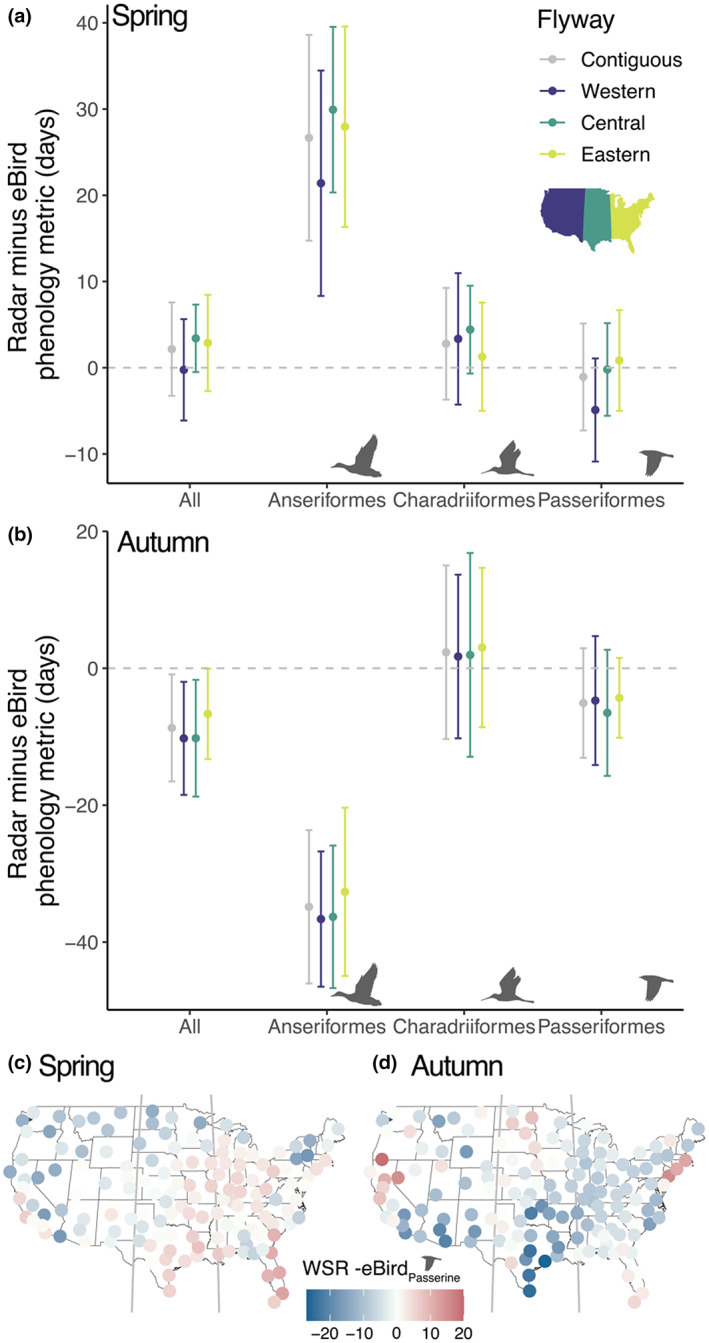
The average difference ± 1 standard deviation between weather surveillance radar (WSR) and eBird‐based estimates of migration phenology during (a) spring and (b) autumn migration (WSR minus eBird) for all 293 species combined and species in three taxonomic orders and WSR stations in three North American migration flyways. Note, scales on the *y* axis differ between (a) and (b). Maps of (c) spring and (d) autumn differences between WSR and eBird‐based estimates of passerine migration phenology. Blue shades show instances of radar showing earlier phenology dates and red shades show instances of eBird showing earlier phenology dates

In addition to examining correlation strength, we quantified the difference in the magnitude of the two phenology indices. For all species combined across the 143 WSR stations, the eBird estimates of migration phenology occurred an average of 2.2 ± 5.4 (± *SD*) days earlier than the WSR estimates during spring migration (Figure 6a). Relative to Anseriformes, spring phenology estimates were more consistent on average in Passeriformes and Charadriiformes, with eBird showing estimates that were −1.1 ± 6.2 (± *SD*) and 2.8 ± 6.5(± *SD*) days earlier than WSR estimates, respectively (Figure 6a). Anseriformes measures were further offset, with an average difference of 26.7 ± 11.9 (± *SD*) days between WSR and eBird estimates (Figure 6a). The overall offset of the phenology estimates was larger during autumn than spring migration, with the WSR estimates of migration phenology averaging 8.7 ± 7.8 (± *SD*) days earlier than eBird across all orders (Figure 6b). Anseriformes dates trailed an average of 34.8 ± 11.2 (± *SD*) days behind WSR, consistent with Anseriformes migration occurring later than the other orders during autumn migration, potentially beyond the WSR sampling period (Figure 6b). Passeriformes were offset by 5.1 ± 8.0 (± *SD*) days on average and Charadriiformes by 2.3 ± 12.7 (± *SD*) days on average (Figure 6b). We found differences across flyways on average during spring and autumn migration in the offset between the WSR and eBird estimates (spring *F*_2,140_ = 5.96, *p* = .003; autumn *F*_2,140_ = 3.76, *p* = .026). Specifically, Tukey HSD post‐hoc tests revealed differences on average in the spring migration offset between the western and central flyways (mean difference = 3.6 days, *p* < .05) and between western and eastern flyways (mean difference = 3.1 days, *p* < .05) – in both cases the western flyway showing smaller offsets. Tukey HSD post‐hoc tests did not reveal pairwise differences during autumn migration. Interestingly, the magnitude of the offset between WSR and passerine‐derived eBird phenology indices varied on average across latitudes of the contiguous United States, becoming more negative (i.e., radar earlier) with increasing latitude during spring migration (β = −0.54, *p* < .001, *R* ^2^ = .24) and increasing with increasing latitude during autumn migration (β = 0.44, *p* < .001, *R* ^2^ = .09) (Figure 6).

## 4 DISCUSSION

In an era of big data, integration of complementary ecological information is critical in describing rapidly changing behaviours and populations, since shortcomings of one source may be overcome by another (Hampton et al., 2013; Isaac et al., 2020). Here, we show a positive correspondence between eBird and WSR migration phenology metrics within the contiguous United States, which could allow the two approaches to illustrate a more complete depiction of the region's avian migration system. Our results indicate that this correlation between migration phenology measured by eBird and WSR was strongest during spring migration. Moreover, phenology metrics showed almost no temporal offset during spring migration [2.2 ± 5.4 (± *SD*) days], although offsets were greater during autumn migration [8.7 ± 7.8 (±SD) days]. While offsets between WSR and Anseriformes‐specific eBird estimates were greatest, estimates derived for Charadriiformes and Passeriformes consistently performed well, suggesting a likely greater contribution of Charadriiformes and Passeriformes to the overall taxonomic make‐up of WSR‐derived phenology estimates.

Using eBird adaptive spatio‐temporal exploratory model (AdaSTEM) estimates of relative abundance, we documented considerable variation in nocturnal migrant species richness across the contiguous United States. Overall, we found higher regional concentrations of nocturnally migratory species during spring migration, with the greatest richness occurring in the Upper‐Midwest. Conversely, the lowest spring richness occurred in the western flyway and the southern portion of the eastern flyway. Looking into the proportions of taxonomic groups, we found Anseriformes generally comprised the smallest proportion of species richness, though richness within the order was greatest at higher latitudes. Unsurprisingly, Passeriformes accounted for the highest proportion of species richness, accounting for over 50% of species richness at 139 of the 143 WSR stations (mean 62.2 ± 6.9%, ± *SD*), with the highest richness occurring within the eastern flyway. Similar patterns held during autumn migration, with Passerines contributing more than 50% of species richness at 137 of the 143 WSR stations (mean 63.2 ± 7.9%, ± *SD*). Of the WSR stations where Passeriformes did not represent the majority, during spring migration, two stations were located along the Pacific coast of the Northwestern United States and the remaining two were located within the interior. During autumn migration, four of the six stations were located along the Pacific coast of the Northwestern United States and the coasts of the Great Lakes (Washington, Michigan, Minnesota). During both seasons, all sites were north of 40°N latitude. Overall, autumn species richness was more evenly distributed and shifted eastward (La Sorte, Fink, Hochachka, DeLong, & Kelling, 2014). While WSR‐derived abundance estimates were not mapped for this study, similar seasonal geographic shifts in migration intensity have been shown by Dokter et al. (2018) and Horton, Van Doren, et al. (2019). These seasonal macroscale patterns are likely driven by the region's prevailing winds (Kranstauber et al., 2015; La Sorte, Fink, Hochachka, Farnsworth, et al., 2014), and by geographic variation in vegetation phenology (La Sorte, Fink, Hochachka, DeLong, & Kelling, 2014; Ng et al., 2022), particularly in the western portion of the continent.

One of the challenges we faced in our assessment of migration phenology was how to properly characterize eBird phenology in a capacity that generalizes to all species. In any one location, species may occur during the breeding or non‐breeding season or in transit during migration. Thus, phenology indices need to be robust to these dynamics. In our approach, we opted to use the date that preceded half of the maximum seasonal abundance (Youngflesh et al., 2021). Via this approach, eBird‐based estimates only slightly preceded those derived from WSR in the spring, but trailed WSR in the autumn, with Anseriformes as the exception. In the case of a true passage migrant, with a diagnostic bell‐shaped passage signature, this extraction approach likely captures the start of migration (i.e., leading edge of passage), rather than the peak (Youngflesh et al., 2021). WSR‐based estimates, in contrast, capture individual birds in active migration (i.e., in flight), so measuring peak passage (i.e., date of max) is more likely a reliable and better approximation of the timing of system‐wide peak activity. Examining the magnitude offsets between WSR and eBird, it is clear that phenology estimates for Anseriformes (waterfowl) are not a primary contributor to those captured by WSR's half‐max passage dates. Because waterfowl tend to migrate early in the spring and late in autumn (La Sorte et al., 2015), it appears that Anseriformes are not as well represented in the WSR signal. The positive correspondence between Passeriformes (songbirds) and Charadriiformes (shorebirds) with WSR phenology estimates indicates their dominance in aerial passage. This outcome suggests that while using all species is valid, focusing on songbirds and shorebirds is sufficient to capture the primary characteristics of passage.

## 5 CONCLUSIONS

This study provided a general comparison of WSR and eBird‐based estimates of migration phenology, identifying key similarities and differences between the two approaches. It would be valuable to estimate these phenology metrics on an annual basis to determine how these two approaches compare and track across years. While WSR data can be used to generate annual estimates, eBird AdaSTEM products currently lack annual replicates. Expansion of these eBird products would have tremendous utility, especially in understanding migrant sensitivity to climate effects (Youngflesh et al., 2021). Additionally, because Passeriformes were shown to be most strongly correlated with radar‐derived timing events, it remains unclear if specific assemblages of migrants within this order could further refine the comparisons. Examining families or even assemblages of common species may reveal dominant signals in macroscale movement patterns that are generalizable to a few species, rather than an entire order. However, uncovering these subsets (e.g., 10 species) presents a non‐trivial task in exploration, as there are many trillions of unique combinations of nocturnal species assemblages (e.g., from 293 total species). Regardless, integration across these two platforms holds promise in depicting a more complete understanding of avian migration, especially regarding important indices, like migration phenology.

## AUTHOR CONTRIBUTIONS

EH and KGH conceived the initial idea for this study, performed statistical analyses, produced figures and results, and drafted the paper; MB provided revisions and insights on radar analyses; FAL and HMM provided additional revisions, eBird insights, and statistical input.

## FUNDING INFORMATION

Funding for this project was provided by National Science Foundation MSB‐NES‐2017554 and ABI sustaining grant: DBI‐1939187, The Wolf Creek Charitable Foundtain,  and National Aeronautics and Space Administration Biodiversity 80NSSC21K1143. NSF GRF #1840343 supported HMM.

## CONFLICT OF INTEREST

We declare we have no competing interests.

## ETHICS STATEMENT

Approval was not required for this study.

## Supporting information


Figure S1

## Data Availability

The weather surveillance radar data generated during and/or analysed during the current study are available at https://doi.org/10.6084/m9.figshare.10062239.v1 and the eBird AdaSTEM abundance data are publicly available: https://ebird.org/st/request.
