# Supplementary material for: The correlation between eBird community science and weather surveillance radar‐based estimates of migration phenology
Source: Glob Ecol Biogeogr. 2022 Jul 13;31(11):2219–30. doi: 10.1111/geb.13567 (PMC9795923; doi:10.1111/geb.13567)
Supplement: Supplementary file 1 — Figure S1 [file GEB-31-2219-s001.docx]

**Supplemental Figure** for *The correlation between eBird community science and weather surveillance radar-based estimates of migration phenology*


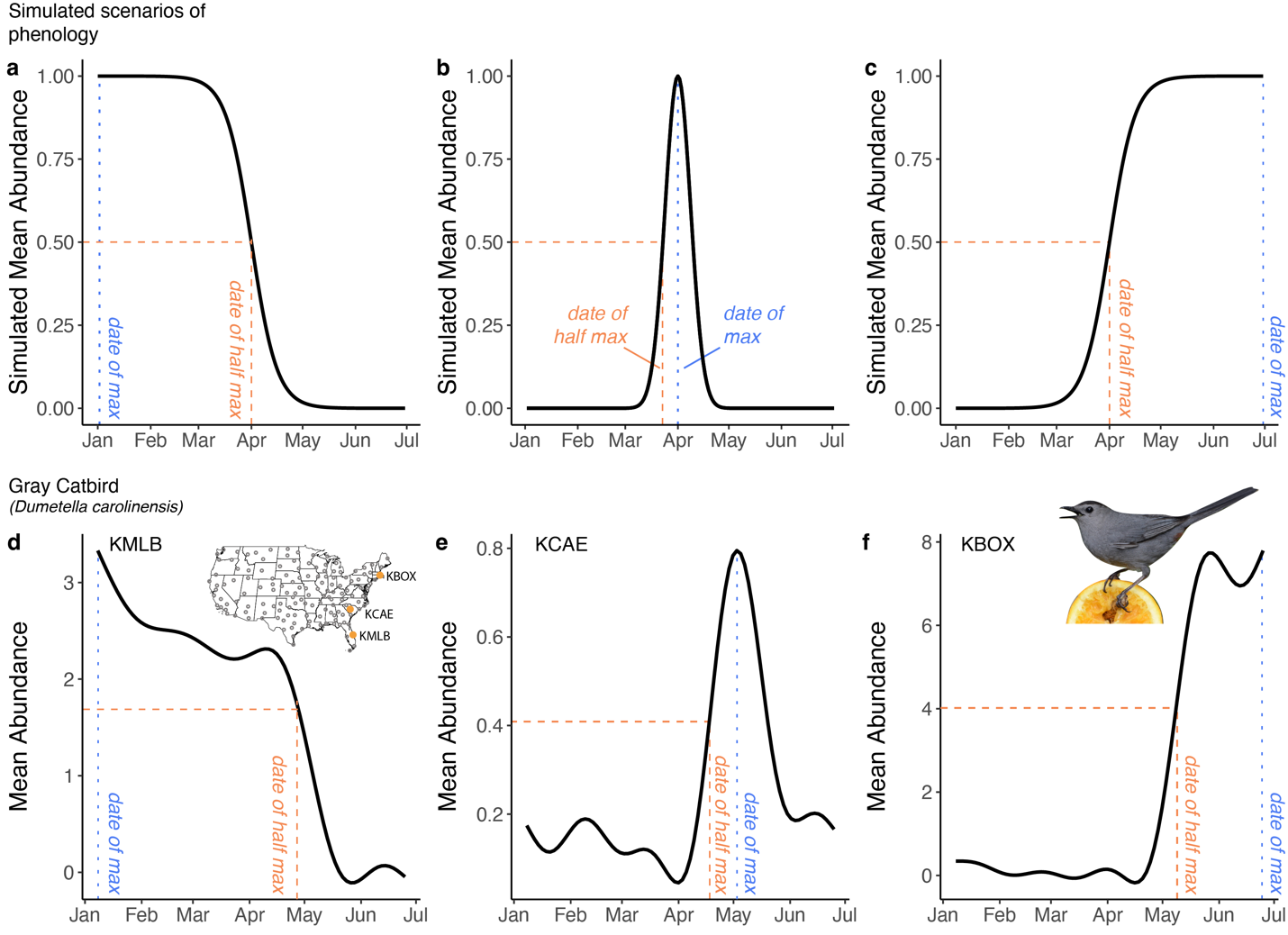


**Figure S1:** We illustrate three possible scenarios for intra-seasonal abundance changes captured by eBird data. In the upper plots, we represent these scenarios conceptually, whereas on the bottom plots we show estimated relative abundance for the Gray Catbird (*Dumetella carolinensis*) in three separate regions of its annual geographic distribution (locations highlighted in the inset map). Figures (a) and (d) show mean abundance throughout the season for species leaving their non-breeding grounds, where initially their abundance is at its peak, and initiating their northward migration. Figures (b) and (e) represent the scenario where species are passing through, with an increase followed by almost equal decrease in mean abundance. Finally, (c) and (f) reflect the case in which species are arriving on their breeding grounds after migration. On each plot, we have highlighted the date of peak mean abundance (in blue) and the date of half maximum mean abundance (in orange), the latter of which being the metric we selected for our analysis.
